# Apathy as a Predictor for Conversion From Mild Cognitive Impairment to Dementia: A Systematic Review and Meta-Analysis of Longitudinal Studies

**DOI:** 10.1177/08919887221093361

**Published:** 2022-04-21

**Authors:** David Fresnais, Mats B. Humble, Susanne Bejerot, Adrian D. Meehan, Brynjar Fure

**Affiliations:** 1 School of Medical Sciences, 6233 Örebro University, Örebro, Sweden; 2 Department of Internal Medicine, Central Hospital Karlstad, Region Värmland, Sweden; 3 Department of Geriatrics, Faculty of Medicine and Health, 6233 Örebro University, Örebro, Sweden

**Keywords:** behavioral disturbance, cognitive impairment, dementia, elderly, apathy

## Abstract

**Background:**

Apathy is one of the most prevalent neurobehavioral manifestations in mild cognitive impairment (MCI) and is included among the behavioral and psychological symptoms of dementia (BPSD). Studies suggest that the presence of apathy could be associated with increased dementia risk. The role of apathy in conversion from MCI to dementia, and whether apathy could be a relevant predictor for dementia progression, are still matters of investigation.

**Aim:**

To study the relationship between apathy and progression to dementia in individuals with MCI.

**Methods:**

A systematic literature search in Medline, Embase, Cochrane Library, Epistemonikos, PsychINFO, and CINAHL was performed according to the Preferred Reporting Items for Systematic Reviews and Meta-Analyses guidelines. The search included longitudinal studies reporting on the association between apathy and dementia.

**Results:**

The main outcome was pooled unadjusted hazard ratios (HR) of apathy in dementia conversion and included 11 studies with 9504 individuals. There was a significant association between apathy and dementia conversion, HR = 1.54; 95% CI, 1.29, 1.84. Subgroup analysis showed a significant association between apathy and progression to AD.

**Conclusion:**

Apathy was associated with an increased risk of conversion to AD and all-cause dementia in patients with MCI. The role of apathy as a marker for incident dementia needs to be investigated in large, high-quality studies.

## Introduction

Mild cognitive impairment (MCI) is a condition characterized by objective evidence of cognitive impairment, sometimes defined as < 1.5 standard deviations (SD) on any cognitive test according to the Petersen criteria, without impairment of activities of daily living.^1^ The Jak & Bondi criteria for MCI require two or more impaired test scores at least one SD below age-normative data.^2^ Individuals with MCI are at increased risk for progressing to dementia, although some remain stable or return to normal cognition.^3,4^ Identifying clinical predictive factors for dementia progression in individuals with MCI is therefore an important clinical concern. Hypothetically, if such clinical predictive factors can be modified by treatment, it could even be explored whether such treatment may delay or prevent conversion to dementia.

In addition to memory loss and other cognitive impairments, neuropsychiatric symptoms are common in both MCI^5^ and dementia.^6^ These behavioral and psychological symptoms of dementia (BPSD) are, in the absence of prominent cognitive impairments, also referred to as mild behavioral impairment (MBI).^7^ The Neuropsychiatric inventory-questionnaire (NPI-Q) is one of several instruments used to identify BPSD and MBI. NPI-Q is a validated, informant-based interview that assesses symptoms in the following 12 domains: agitation, irritability, disinhibition, elation, motor disturbance, depression, anxiety, apathy, sleep, appetite, delusions, and hallucinations.^8^ One of the most prevalent behavioral symptoms in MCI and dementia is apathy,^9,10^ which can be defined as a loss of motivation in conjunction with reduced goal-directed behavior, cognitive activity and emotions.^11^ Apathy and apathy symptoms, in both clinical practice and research settings, can be assessed and defined using different criteria and instruments. In addition to the NPI-Q, apathy can be assessed using the Apathy Evaluation Scale (AES)^12^ or a subscale of the Geriatric Depression Scale (GDS),^13^ among others. Apathy has been shown to increase the risk of dementia progression in individuals with MCI.^14-16^ Apathy has also been correlated with higher levels of neurofibrillary tangles in individuals with dementia.^17^ A previous meta-analysis including studies published before October 2017 showed that apathy almost doubled the risk of progression from MCI to dementia in memory clinic patients^18^ and, since then, several studies on this topic have been published. Apathy has therefore been proposed as a potential prognostic factor for impending dementia.

The role of apathy in predicting progression from MCI to dementia has been further explored in recent longitudinal studies which present diverging results,^19-21^ with varying incidence of dementia conversion possibly due to differences in follow-up time, cognitive instruments used and sex distribution among studies, and is still a matter of investigation. The present meta-analysis was performed to evaluate the evidence from longitudinal cohort studies for the potential association between apathy in individuals with MCI and risk of incident dementia.

## Methods

### Search Strategy

#### Searches

The predefined review protocol for this systematic review and meta-analysis was registered in the International Prospective Register of Systematic Reviews (PROSPERO) (CRD42021274742). Medline, EMBASE, Cochrane Library, PsycINFO, CINAHL, and Epistemonikos were systematically searched from 2000 to 2021 to identify longitudinal cohort studies assessing apathy and subsequent incident dementia. The search strategy included the following pre-specified criteria: (1) individuals with MCI; (2) assessment of BPSD including apathy; (3) clearly defined dementia diagnosis; (4) longitudinal studies. Search terms included “cognitive dysfunction,” “mild cognitive impairment,” “dementia,” “Alzheimer’s disease,” “behavioral and psychological symptoms of dementia,” “conversion,” “progression,” “prospective studies,” and “longitudinal studies.” The search was performed with the help of a trained information specialist and was limited to English or Scandinavian language studies. The full search strategy is shown in Supplementary Material 1.

### Selection Criteria

The present systematic review followed the Preferred Reporting Items for Systematic Reviews and Meta-analyses (PRISMA). Two investigators applied eligibility criteria and independently screened titles and abstracts for inclusion after the removal of duplicated literature. Full-text records were then assessed according to the pre-specified selection criteria. Included studies had to report on the association between apathy and dementia diagnoses in individuals with MCI. Studies focusing on patients selected for specific medical conditions such as stroke or other neurological conditions were excluded. One additional study was identified from hand searching reference lists in previous reviews regarding the association between apathy and incident dementia.

### Data Extraction and Analysis

Two investigators independently extracted data from the included studies. HRs and ORs for conversion from MCI to dementia in patients with symptoms of apathy were obtained, as well as measurements of apathy, ascertainment of MCI and dementia diagnoses, criteria for conversion to dementia, and subtype of dementia. Since different studies had adjusted HRs and ORs for different variables, we chose to use unadjusted HRs and ORs in our meta-analyses to avoid introducing more heterogeneity in our results. Information on the general characteristics of the included studies was obtained according to a data extraction form, including: first author of the study, publication year, country and setting of research, study design, number of patients included, and patient demographics including age, sex distribution, and educational level of included patients. Study investigators were not contacted for unreported data or additional details. If data on different subtypes of dementia was available, all-cause dementia was used. A subgroup analysis using AD as the preferred outcome was carried out.

### Statistical Methods

The risk of conversion from MCI to dementia in patients with symptoms of apathy was explored using HRs and ORs in logarithmic scales. Weights were automatically assigned according to the algorithm in RevMan 5.4.1.^22^ Differences in the proportion of individuals with apathy among converters to dementia compared to non-converters were also analyzed. Additionally, mean apathy scores according to the NPI were compared between converters and non-converters. Random-effects models were used due to the heterogenous study characteristics. P-values were 2-sided.

Statistical analyses were performed using RevMan 5.4.1.^22^

### Quality Assessment and Risk of Bias

The quality of the included studies, including risk of bias in each study, was assessed independently by two investigators using the Critical Appraisals Skills Programme (CASP)^23^ checklist designed for cohort studies. The Grading of Recommendations, Assessment, Development and Evaluations (GRADE)^24^ tool was used to evaluate the overall confidence in the effect estimates across studies for each pooled outcome.

### Disagreements Between Individual Judgments

Any disagreements in study selection, data extraction or quality assessment of studies were resolved by consensus.

## Results

### Literature Search

**Figure 1.**
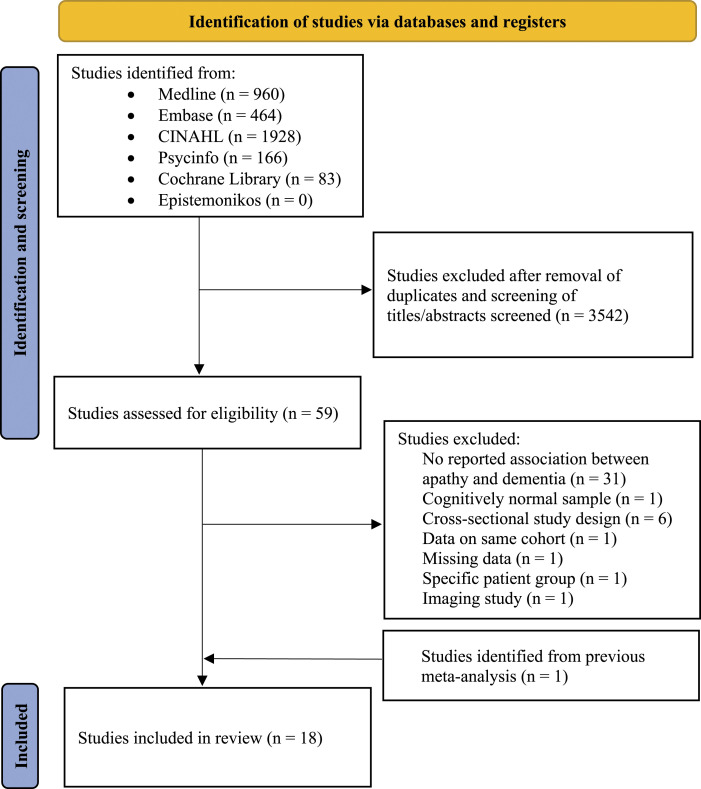
PRISMA 2020 flow chart showing process of identification of studies. PRISMA = Preferred Reporting Items for Systematic Reviews and Meta-analyses, n = number.

A total of 18 relevant studies were identified from the literature search after removal of duplicated and irrelevant literature and screening titles and abstracts.^14,19-21,25-38^ In all, 59 articles were read in full text for eligibility, and of these, 17 studies fulfilled the pre-specified inclusion criteria. One additional article was identified from a previously published meta-analysis.^29^ The process of finding relevant articles is demonstrated in Figure 1; Supplementary Material 2 shows the reasons for the exclusion of 42 articles after full-text reading.

### Study Characteristics

Table 1 shows the general characteristics of included studies, ascertainment of MCI, and measurement and proportion of individuals with apathy. Dementia subtype and diagnostic criteria, conversion rates from MCI to dementia, duration of follow-up, and the reported association between apathy and dementia conversion are shown in Table 2. All studies were prospective cohort studies and published between 2009 and 2020. Conversion rates from MCI to dementia ranged from 15.2^14^ to 41.9%.^37^ The proportion of individuals presenting with apathy was between 9.2 and 65.0%. Mean follow-up time was 3.1 years. The most frequently used diagnostic criteria for MCI and dementia were the Petersen criteria^1^ and NINCDS-ADRDA^39^ followed by DSM-IV.^40^ Measurements of apathy varied across studies and NPI-Q^8^ was the most commonly used instrument, defining apathy as either mild (score = 1), moderate (score = 2), or severe (score = 3).

**Table 1.** General characteristics of included studies and ascertainment of cognitive impairment and apathy.

Author and year of publication	Country	Setting	Sample and diagnostic criteria for MCI	N	Age, N (SD)	Female, N, (%)	Education, mean (SD), y	Tool used for measurement of apathy; and cases, N (%)
Yang,^20^ 2020	China	Population-based	MCI, ≥ 60 years; compound memory score ≤1.5 SD compared to NCI	465	With NPS 76.3 (6.6) years; no NPS 75.3 (7.4)	With NPS (72.8); no NPS (66.1)	NPS 3.8 (3.2); no NPS 2.6 (3.1)	Chinese version of the NPI-Q (CNPI-Q); 59 (12.7)
Mallo,^24^ 2020	Spain	Health centers	MCI/NCI; Petersen criteria	128	67.3 (8.0)	74 (57.8)	NR	NPI-Q, NR
Dietlin,^19^ 2019	France	Memory clinic	MCI; MMSE ≥24	96	78.7 (5.6)	57 (59.4)	8.4 (3.9)	NPI-Q, NR
Goukasian,^25^ 2019	USA	Database of memory clinics	MCI, SMC, NCI, AD; ADNI criteria	1077; 559 MCI	71.9 (7.4)	240^43^	16.1 (2.7)	NPI, NPI-Q; 170 (30.4)
Ruthirakuhan,^18^ 2019	Canada	Database of memory clinics	MCI; NR	4932	73.2 (9.2)	2450 (49.6)	Apathy 15.4 (3.2); no apathy 15.3 (3.3)	NPI-Q; 8% apathy only, 14% apathy and depression
Yokoi,^26^ 2019	Japan	Database of memory clinics	MCI; (i) memory disturbance confirmed by study partner; (ii) MMSE score >23; (iii) CDR score of .5; and (iv) logical memory on WMS-R	234	72.8 (5.9)	118 (50.4)	13.0 (2.8)	NPI-Q (translated and validated in Japanese); 43 (18.5)
Guercio,^27^ 2015	United States	Memory clinic	MCI/NCI; no dementia, preserved ADL, Logical memory IIa on WMS-R, CDR score of .5, MMSE >23	75; 57 MCI	74.7 (8.0)	31 (41.3)	16.8 (2.6)	AES; NR
Pink,^28^ 2015	United States	Population-based	MCI; revised Mayo Clinic criteria for MCI	322	82.1 (IQR 77.7, 85.0)	151 (45.5)	12 (IQR 12, 15)	NPI-Q; 55 (16.6)
Brodaty,^29^ 2014	Australia	Memory clinics	MCI; Petersen criteria	185	Dementia 75.3 (6.7); no dementia 76.0 (7.0)	Dementia 29 (55.8); no dementia 56 (42.1)	Dementia (46.2%); no dementia (50.0%)	NPI; 59 (31.9)
Sobów,^30^ 2014	Poland	Memory clinic	MCI; NIA-AA, CDR score of 0.5	83	75.0 (1.9)	33 (56.9)	9	NPI; 20 (24.1)
Peters,^31^ 2013	United States	Population-based	CIND; 1.5 SD < normative mean, CDR score of .5	230	81.9 (5.1)	115 (50.0)	13.4 (3.1)	NPI; 21 (9.2)
Rosenberg,^32^ 2013	United States	Database of memory clinics	MCI; clinical diagnosis	1821	75.3 (9.3)	920 (50.5)	15.2 (6.2)	NPI-Q, GDS; 304 (16.7)
Somme,^33^ 2013	Spain	Outpatient memory unit	a-MCI; adapted operational criteria for amnestic MCI	132	69.8 (8.7)	62 (46.9)	10.6 (4.4)	Spanish 12-item version of the NPI; 69 (52.2)
Richard,^34^ 2012	Netherlands	Database of memory clinics	MCI; WMS-R, MMSE >23, and CDR >0.5	397	74.8 (7.5)	141 (35.5)	15.7 (3.0)	GDS-3A; 178 (44.8)
Gallassi,^35^ 2010	Italy	Memory clinic	MCI/NCI; Neuropsychological tests with 1.5-SD cutoff derived by the same normative data	92;54 included in analyses	68.9 (8.9)	33 (61.1)	8.7 (4.7)	NPI; NR
Palmer,^13^ 2010	Italy	Memory clinic	a-MCI; Petersen criteria	131	70.8 (6.5)	54 (41.2)	High education 80 (61.1%)	NPI, clinical, Starkenstein criteria; 14 (10.7)
Ramakers^36^, 2009	Netherlands	Memory clinic	MCI; Petersen criteria	263	66.9 (7.7)	116^44^	Low, middle, high (%): 23/46/31	HAMD; 171 (65.0)
Vicini Chilovi,^37^ 2009	Italy	Memory clinic	MCI; (i) presence of a cognitive complaint; (ii) absence of dementia; (iii) change from normal functioning; (iv) decline in any area of cognitive functioning; (v) preserved overall functioning	124	71.3 (7.7)	84 (68)	7.4 (3.5)	AES; 36 (29.0)

NR = not reported; SD = standard deviation; SMC = subjective memory complaint; MCI = mild cognitive impairment; a- MCI = amnestic-mild cognitive impairment; n = number; y = years; NCI = no cognitive impairment; NPI = Neuropsychiatric Inventory; NPI-Q = Neuropsychiatric Inventory-Questionnaire; MMSE = Mini-Mental State Examination; ADNI = Alzheimer’s Disease Neuroimaging Initiative; CDR = Clinical Dementia Rating; WMS-r = Wechsler Memory Scale-revised; ADL = Activities of Daily Living; AES = Apathy Evaluation Scale; IQR = Interquartile range; NIA-AA = the National Institute of Aging and Alzheimer’s Association; CIND = Cognitive Impairment No Dementia; GDS = Geriatric Depression Scale; HAMD = Hamilton Depression Rating Scale.

**Table 2.** Association between apathy and dementia, follow-up, and dementia incidence.

Author and year of publication	Dementia subtype	Dementia diagnostic criteria	Follow-up time, mean (SD), y	Conversion to dementia		Reported association between apathy and dementia conversion (95% CI), unadjusted
				Overall, N (%)	Apathy, N (%)	
Yang,^20^ 2020	AD	DSM-IV-TR criteria	3	78 (16.8)	5 (7.9)	OR: .44 (.08–2.42)
Mallo,^24^ 2020	All-cause	DMS-4-TR	6*	30 (23.4)	NR	NR
Dietlin,^19^ 2019	AD	CDR	3.5 (IQR: 2.9-4)	42 (43.8)	NR	HR: 1.54 (.78–3.04)
Goukasian,^25^ 2019	AD	NINCDS-ADRDA	3.6 (2.1)	NR	NR	HR: 3.02 (2.17–4.20)
Ruthirakuhan,^18^ 2019	AD	NINCDS-ADRDA, NIA-AA	1.9	1713 (34.7)	NR	HR: 1.24 (1.05–1.46)
Yokoi,^26^ 2019	AD	NINCDS-ADRDA	3.5 (.8)	101 (43.2)	24 (23.8)	HR: 1.57 (.99–2.49)
Guercio,^27^ 2015	AD	CDR	MCI: 1.4 (1.2) NCI: .7 (.7)	11 (28.2)	NR	HR: 1.19 (1.09–1.3)
Pink,^28^ 2015	All-cause	DSM-IV	3.0 (IQR: 2.5–5.3)	117 (35.2)	25 (45.5)	HR: 1.62 (1.03–2.55)
Brodaty,^29^ 2014	All-cause	DSM-IV	3	52 (28.1)	NR	NR
Sobów,^30^ 2014	All-cause	Change in CDR score from .5 to 1 (or more)	2	27 (32.5)	18 (90.0)	OR: 70.7 (5.70–876.9)
Peters,^31^ 2013	All-cause	NINCDS-ADRDA; NINDS-AIREN	3.3	85 (37.0)	7 (33.3)	HR: .93 (.43–2.01)
Rosenberg,^32^ 2013	All-cause, AD	CDR, clinical diagnosis	1.2 (.3)	NR	NR	HR: 1.38 (1.2–1.56)
Somme,^33^ 2013	AD, all-cause	DSM-IV criteria for dementia and NINCDS-ADRDA criteria for AD	3.5 (2.9)	38 (28.8)	21 (30.4)	HR: 2.2 (1.00–4.82)
Richard,^34^ 2012	All-cause	Clinical	2.7 (1.0)	166 (41.8)	82 (46.1)	HR: 1.43 (.86–2.38)
Gallassi,^35^ 2010	All-cause	DSM-IV	4	10 (18.5)	NR	NR
Palmer,^13^ 2010	AD	NINCDS-ADRDA	1.4 (8.4)	15 (15.2)	6^50^	HR: 4.6 (1.70–12.45)
Ramakers,^36^ 2009	AD, all-cause	NINCDS-ADRDA and DSM-IV	5.4	90 (41.9)	50 (37.0)	OR: .67 (.4–1.12)
Vicini Chilovi,^37^ 2009	All-cause	NINCDS-ADRDA, NINDS-AIREN	2.0 (.2)	28 (22.6)	13 (36.1)	7.07 (1.99–25.12)

SD = Standard deviation; y = years; CI = confidence interval; n = number; DSM-IV = Diagnostic and Statistic Manual of Mental Disorders fourth edition; DSM-IV-TR = Diagnostic and Statistic Manual of Mental Disorders fourth edition-text revision; OR = odds ratio; HR = hazard ratio; CDR = Clinical Dementia Rating; AD = Alzheimer’s disease; IQR = interquartile range; NINCDS-ADRA = National Institute of Neurological and Communicative Diseases and Stroke/Alzheimer’s Disease and Related Disorders Association; NIA-AA = the National Institute of Aging and Alzheimer’s Association; NINDS-AIREN = ; National Institute of Neurological Disorders and Stroke and the Association Internationale pour l’Enseignement en Neurosciences.

“up to 6 years follow-up”.

### Meta-Analysis of Results

The association between apathy and conversion from MCI to dementia was assessed by analyzing unadjusted HRs of apathy, if available. Additional analyses of ORs for apathy in conversion to dementia, mean NPI scores and the proportion of individuals with apathy between converters and non-converters were performed. Moreover, a subgroup analysis of HRs of apathy in conversion from MCI to AD was performed.

#### HRs of Apathy in Conversion from MCI to AD and All-Cause Dementia

**Figure 2.**
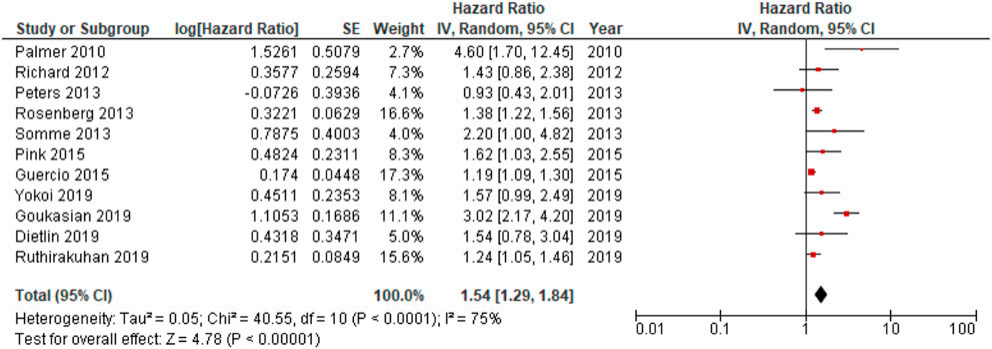
Forest plot showing HR of apathy in conversion from MCI to all-cause dementia; MCI = Mild Cognitive Impairment; CI = Confidence Interval; IV = Inverse Variance.

**Figure 3.**
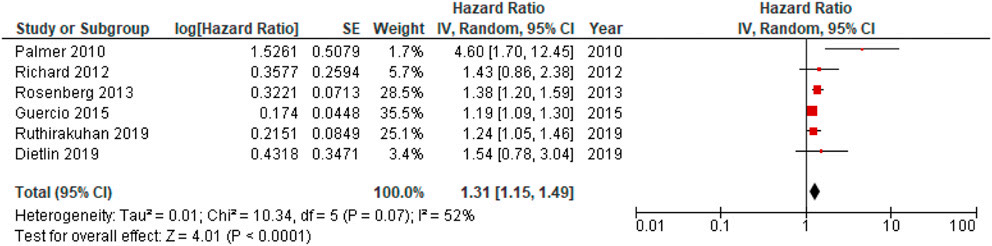
Forest plot showing HR of apathy in conversion from MCI to AD; AD = Alzheimer’s Disease; MCI = Mild Cognitive Impairment; CI = Confidence Interval, IV = Inverse Variance.

Eleven out of 18 studies included in the meta-analyses were used in the main analysis of HRs for apathy in conversion to dementia, and involved 9504 individuals.^14,19,20,26–29,32–35^ Sample sizes of included studies ranged from 83^31^ to 4932.^19^ Meta-analysis of pooled HRs for apathy in dementia conversion showed a statistically significant association between apathy and dementia conversion, HR = 1.54; 95% CI, 1.29, 1.84; P < .001, see Figure 2. In addition, analysis of HRs for apathy in conversion to AD revealed a significant association,HR = 1.31; 95% CI, 1.15, 1.49; P < .001, see Figure 3.

#### Additional Analyses; Proportion of Individuals with Apathy, Mean NPI-Scores, ORs of Apathy

**Figure 4.**
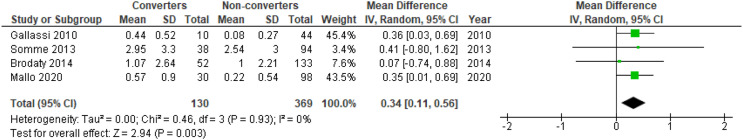
Forest plot showing mean difference in NPI apathy scores between converters from MCI to dementia versus non-converters; MCI = Mild Cognitive Impairment; NPI = Neuropsychiatric Inventory; CI = Confidence Interval, IV = Inverse Variance.

**Figure 5.**
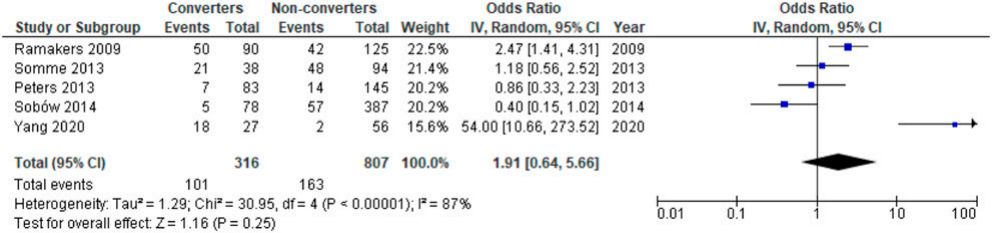
Forest plot showing the proportion of individuals with apathy symptoms in converters from MCI to dementia versus non-converters; MCI = Mild Cognitive Impairment; CI = Confidence Interval, IV = Inverse Variance.

**Figure 6.**
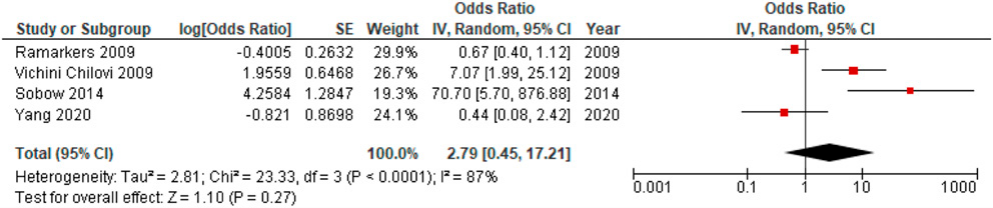
Forest plot showing OR of apathy in conversion from MCI to all-cause dementia; MCI = Mild Cognitive Impairment; CI = Confidence Interval, IV = Inverse Variance.

There was a significant difference in mean NPI apathy scores between converters and non-converters with mean difference (MD) = .34; 95% confidence interval, .11, .56; P = .003, but not for the proportion of individuals with apathy in converters compared to non-converters, OR = 1.91; 95% CI, .64, 5.66; P = .25, see Figure 4 and Figure 5, respectively. Meta-analysis of ORs for apathy in dementia conversion showed no significant association, OR = 2.79; 95% CI, .45, 17.21; P = .27, see Figure 6.

### Assessment of Quality of the Included Studies

Table 3 shows the evaluation of included studies according to the CASP checklist for cohort studies.

**Table 3.** Assessment according to CASP (Q 1-5, 9-11 for cohort studies) checklists for included studies.

Author and year of publication	Did the study address a clearly focused issue?	Was the cohort recruited in an acceptable way?	Was the exposure accurately measured to minimize bias?	Was the outcome accurately measured to minimize bias?	Have the authors identified all important confounding factors?	Have they taken account of the confounding factors in the design and/or analysis?	Was the follow-up of subjects complete enough?	Was the follow-up of subjects long enough?	Do you believe the results?	Can the results be applied to the local population?	Do the results fit with other available evidence?	Overall appraisal
Yang, 2020	+	?	+	+	+	+	+	+	+	+	+	?
Mallo, 2020	+	?	+	+	—	—	?	+	+	+	+	—
Dietlin, 2019	+	+	+	+	+	—	+	+	+	+	+	—
Ruthirakuhan, 2019	+	+	+	+	+	+	?	?	+	+	+	?
Goukasian 2019	+	+	+	+	+	+	+	+	+	+	+	+
Yokoi 2019	+	?	+	+	+	+	+	+	+	+	+	+
Guercio, 2015	+	+	+	+	+	+	+	+	+	+	+	+
Pink, 2015	+	+	+	+	+	+	+	+	+	+	+	+
Brodaty, 2014	+	+	+	+	+	+	-	+	+	+	+	—
Sobów, 2014	+	+	+	+	+	+	?	+	+	+	+	?
Peters, 2013	+	?	+	+	+	+	+	+	+	+	+	?
Rosenberg, 2013	+	?	+	?	+	+	-	+	+	+	+	—
Somme, 2013	+	+	+	+	+	+	+	+	+	+	+	+
Richard, 2012	+	?	+	?	+	+	?	+	+	+	+	?
Gallassi, 2010	+	?	+	+	+	+	—	+	+	+	+	—
Palmer, 2010	+	++	+	+	+	+	+	+	+	+	+	+
Ramakers, 2009	+	?	+	+	+	+	+	+	+	+	+	?
Vicini Chilovi, 2009	+	?	+	?	+	—	?	+	+	+	+	—

CASP = Critical Appraisals Skills Programme; +, yes; -, no; ?, cannot tell; Q = question (s).

GRADE was used for the assessment of confidence in the pooled estimates, which was estimated as low to very low, meaning that there is a significant possibility that further studies will affect the results; see Table 4.

**Table 4.** GRADE evidence table with assessments of our confidence in the estimates.

Certainty assessment							№ of patients		Effect		Certainty	Importance
№ of studies	Study design	Risk of bias	Inconsistency	Indirectness	Imprecision	Other considerations	Apathy	No apathy	Relative(95% CI)	Absolute (95% CI)		
HR of apathy in conversion from MCI to all-cause dementia												
11	observational studies	serious^a,b,c^	not serious	not serious	not serious	strong association			—	1.54 HR higher (1.29 higher to 1.84 higher)	⨁⨁○○ Low	IMPORTANT
HR of apathy in conversion from MCI to AD												
6	observational studies	serious^a,b,c^	not serious	not serious	not serious	strong association			—	HR 1.31 higher (1.15 higher to 1.49 higher)	⨁⨁○○ Low	IMPORTANT
Mean difference in NPI apathy score between converters from MCI to dementia versus non-converters												
4	observational studies	serious a,b,c	not serious	not serious	serious d,e	none			-	MD .34 NPI points higher (.11 higher to .56 higher)	⨁○○○Very low	IMPORTANT
Proportion of individuals with apathy symptoms in converters from MCI to dementia versus non-converters												
5	observational studies	serious a,b,c	serious d,f	not serious	serious d,e	none			—	OR 1.38 higher (.47 higher to 4.07 higher)	⨁○○○ Very low	IMPORTANT
OR of apathy in conversion of MCI to all-cause dementia												
4	observational studies	serious^a,b,c^	serious^d,f^	not serious	serious^d,e^	none			—	OR 2.78 higher (.45 higher to 17.08 higher)	⨁○○○ Very low	IMPORTANT

CI: Confidence interval; MD: Mean difference; HR = Hazard Ratio; OR = Odds Ratio; AD = Alzheimers’s Disease; MCI = Mild Cognitive Impairment; NPI = Neuropsychiatric Index GRADE = Grading of Recommendations, Assessment, Development and Evaluations.

^a^Unclear attrition bias.

^b^Inclusion of participants

^c^Unclear if confounders were considered in analyses

^d^Wide CI

^e^Few participants in some studies

^f^Crosses line of no effect.

## Discussion

This systematic review and meta-analysis was conducted in order to investigate the role of apathy in conversion from MCI to dementia. Analyses of HRs of incident dementia revealed a significant relationship between apathy and dementia conversion. The association with apathy was statistically significant for both all-cause dementia and AD, although confidence in our pooled estimates is “low” according to GRADE. Further analyses of NPI-apathy scores and the proportion of individuals with apathy in converters versus non-converters to dementia supported this association, with higher scores and presence of symptoms, respectively, in converters compared to non-converters. On the other hand, when performing analyses of ORs extracted from the included studies, this association disappeared.

Results appear generalizable to memory clinic patients but may not be directly applicable to older community-dwelling individuals, since most studies were performed in a memory clinic setting. The number of patients presenting with apathy symptoms at baseline and the conversion rates from MCI to dementia are in line with what is reported in the literature.^41^ Most studies used standard diagnostic criteria for MCI and dementia although diagnostic criteria varied between studies. The mean follow-up time was 3.1 years. Since the annual progression from MCI to dementia is approximately 10% in clinical settings,^42^ a longer follow-up time would have allowed for the identification of subjects who progressed to dementia at a later stage.

A number of studies have explored the relationship between apathy and dementia conversion in individuals with MCI, most of them reporting apathy to be associated with incident dementia.^14,19,26,28,29,33^ However some studies did not find apathy to be a risk factor for dementia conversion.^20,27,32,35^ Disparities in results may be a consequence of differences in study design, as well as definitions of apathy, MCI, and dementia. MCI was most commonly diagnosed using the Petersen criteria^1^ or modified versions of these criteria, and the most frequently used diagnostic criteria for dementia were DSM-IV^40^ and NINCDS-ADRDA.^39^ While apathy was assessed using NPI-Q^8^ in the majority of studies, the cut-off values for diagnosis of apathy varied in some studies. It is possible that the NPI was used since it is convenient for detecting other neuropsychiatric symptoms in addition to apathy. There are currently very few other available apathy instruments in clinical practice. In the study by Brodaty et al., the NPI-Q cut-off for apathy was at least 1 point,^30^ while the cut-off was at least 2 points in the study by Palmer et al.^14^ In addition, some studies looked at specific symptoms of apathy instead of using NPI-Q. The meta-analysis by van Dalen et al.^18^ from 2018 found a two-fold increased risk for incident dementia in MCI patients with apathy.^18^ This meta-analysis included studies until 2017 and since then several new publications on the subject have appeared. Among the more recent publications (published 2019 or later), Ruthirakuhan et al.^19^ and Goukasian et al,^26^ found a significant association for dementia conversion while Dietlin et al*.*^20^ and Yokoi et al.^27^ did not, although a non-significant trend towards dementia conversion was observed.

Subgroup analyses revealed that apathy was associated with higher risk of conversion from MCI to AD, in addition to all-cause dementia. This was expected since mixed pathologies of vascular disturbances and neurodegeneration are common in AD, especially in elderly individuals.^43^ Indeed, Palmer 2010 found that apathy was associated with a sevenfold increased risk of incident AD in patients with amnestic MCI.^14^

The biological mechanisms by which apathy and other neurobehavioral manifestations may contribute to incident dementia in MCI is currently a matter of investigation, and it is not clear whether apathy is merely prodromal to dementia or a causal risk factor. One study found that elderly individuals with symptoms of apathy had reduced brain volumes compared to those without apathy,^44^ and it is therefore plausible that apathy could indicate changes and disease processes associated with cognitive disorders. It is well known that apathy can be a symptom of depression.^12^ Several of the included studies excluded individuals with a diagnosis of major depressive disorder. One study, which compared the contribution of apathy and depression to dementia conversion, found that apathy alone, but not depression alone, significantly increased the risk of conversion from MCI to dementia,^19^ indicating that apathy could be linked to neuropathological processes in the brain distinct from those of depression. Apathy in individuals with MCI could increase the risk of dementia conversion through neurodegenerative pathology. Indeed, the presence of apathy has been independently associated with AD pathology,^45^ brain hypo-metabolism,^46^ and alterations in neurotransmitter systems in patients with cognitive impairment.^47^ In individuals with MCI, apathy could indicate an underlying neuropathology, and that these patients are at increased risk of incident dementia.^48^ The fact that apathy and other neuropsychiatric symptoms can represent underlying neurodegenerative and vascular pathology, as seen in dementia, has led to the concept of “Mild Behavioral Impairment,” which is a term for individuals presenting with neuropsychiatric symptoms without evidence of concurrent cognitive impairment, used to identify individuals at higher risk for dementia.^49^

However, a statistically significant association between apathy and incident dementia does not necessarily confer a clinical significance, and the potential use of apathy in predicting conversion to dementia in MCI patients is limited by the lack of validated and adapted tools for measuring apathy in MCI. In addition, apathy is a non-specific symptom occurring in numerous other disease processes affecting the brain, such as in multiple sclerosis,^50^ Parkinson’s disease,^51^ post stroke,^52,53^ psychosis,^54,55^ and depression,^12^ and in hormonal disturbances such as hypothyroidism^56^ and hyperparathyroidism,^57^ all of which occur increasingly with age. Apathy is also common in elderly individuals without cognitive impairment,^58^ and increases with age in otherwise healthy community-dwelling individuals.^30^

The analysis of mean NPI apathy scores revealed a significant mean difference of .34 points, which is a very modest difference in a clinical setting. Still, previous studies report that even mild symptoms of apathy and other neuropsychiatric symptoms increase the risk of progression from MCI to dementia, with potential importance in clinical practice. Palmer et al.^14^ demonstrated that milder symptoms of apathy measured with NPI could predict progression to AD, even without a concurrent apathy diagnosis.

Studies included in the current meta-analysis used different tools to assess apathy, most frequently NPI. Guercio et al.^28^ found that apathy scores reported by clinicians were more useful in predicting conversion to dementia than scores reported by informants or subjects. It has been shown that it is challenging for individuals with MCI accurately to report cognitive and behavioral symptoms, most likely due to anosognosia or reduced memory. This is supported by the study by Sugarman et al*.* reporting significant associations for symptoms measured with NPI-Q, which is informant based, but not for GDS-15, when used as a self-reported scale.^48^ Therefore, apathy assessed by clinicians might be more useful in predicting conversion from MCI to dementia. In clinical situations, instruments for apathy diagnosis such as AES^12^ could be considered in the investigation of dementia as well as depression in elderly patients. This would allow for close follow-up of individuals with combined apathy and cognitive impairment who are at excess risk of disease progression. In addition, these patients may profit from potential new apathy treatment options. Indeed, a recently published RCT found positive effects of methylphenidate up to 6 months compared to placebo for the treatment of apathy symptoms in patients with AD.^59^

There are some limitations in the current review and meta-analysis: (1) We did not include conference abstracts in the article selection process or contact authors in cases of missing data, and therefore some literature may have been overlooked. (2) Since apathy and progression to dementia are associated with study dropout, there is risk of attrition bias and attenuation of results. (3) HRs were not available for all studies. (4) Analyses were not always adjusted for symptoms of depression. (5) The confidence in our pooled estimates according to GRADE are “low” or “very low” due to methodological weaknesses which included, most frequently, missing information on inclusion criteria, small sample sizes in some studies, unclear attrition bias and wide CIs; (6) some of the analyses included relatively few studies; (7) ORs used in two of our meta-analyses might have been more prone to attrition bias than HRs. Nevertheless, this systematic review and meta-analysis presents a transparent methodology of sorting, including and assessing studies, and accuracy of the literature search was ensured by a trained information specialist.

In conclusion, the results from the current meta-analysis suggest that apathy is associated with an increased risk of conversion from MCI to all-cause dementia as well as to AD. However, no firm conclusion regarding the role of apathy in dementia progression can be established due to the methodological weaknesses in many of the included studies that affect our confidence in the results. The role of apathy in predicting incident dementia, alone or in combination with other neuropsychiatric symptoms and clinical parameters, needs to be investigated in larger, high-quality studies. Further studies should also preferably include the concept of MBI.

## Supplemental Material

Supplemental Material - Apathy as a Predictor for Conversion From Mild Cognitive Impairment to Dementia: A Systematic Review and Meta-Analysis of Longitudinal Studies Click here for additional data file. Supplemental Material for Apathy as a Predictor for Conversion From Mild Cognitive Impairment to Dementia: A Systematic Review and Meta-Analysis of Longitudinal Studies by David Fresnais, Mats B. Humble, Susanne Bejerot, Adrian D. Meehan, and Brynjar Furein Journal of Geriatric Psychiatry and Neurology

Supplemental Material - Apathy as a Predictor for Conversion From Mild Cognitive Impairment to Dementia: A Systematic Review and Meta-Analysis of Longitudinal Studies Click here for additional data file. Supplemental Material for Apathy as a Predictor for Conversion From Mild Cognitive Impairment to Dementia: A Systematic Review and Meta-Analysis of Longitudinal Studies by David Fresnais, Mats B. Humble, Susanne Bejerot, Adrian D. Meehan, and Brynjar Furein Journal of Geriatric Psychiatry and Neurology

## Appendix

### Abbreviations

AD: Alzheimer’s disease;
AES: Apathy Evaluation Scale
BPSD: Behavioral and Psychological Symptoms of Dementia;
CASP: Critical Appraisals Skills Programme;
CDR: Clinical Dementia Rating;
CI: Confidence Interval;
CIND: Cognitive Impairment No Dementia;
DSM-IV: Diagnostic and Statistical Manual of Mental Disorders 4th edition;
DSM-4-TR: Diagnostic and Statistical Manual of Mental Disorders 4th edition, text revised;
GDS: Geriatric Depression Scale;
GRADE: The Grading of Recommendations, Assessment, Development, and Evaluations;
HAM-D: The Hamilton Depression Rating Scale;
HR(s): Hazard Ratio(s);
IV: Inverse Variance;
MBI: Mild Behavioral Impairment;
MCI: Mild Cognitive Impairment;
NCI: No Cognitive Impairment;
MD: Mean Difference;
NIA-AA: National Institute on Aging and Alzheimer’s Association;
NPI: Neuropsychiatric Inventory;
NPI-Q: Neuropsychiatric Inventory Questionnaire;
NINCDS-ADRDA: National Institute of Neurological and Communicative Diseases and Stroke/Alzheimer’s Disease and Related Disorders Association;
NINDS-AIREN: National Institute of Neurological Disorders and Stroke and Association Internationale pour la Recherché et l'Enseignement en Neurosciences;
OR(s): Odds Ratio(s);
PRISMA: Preferred Reporting Items for Systematic Reviews and Meta-analyses;
PROSPERO: International Prospective Register of Systematic Reviews;
SD: Standard Deviation;
WMR: Wechsler Memory Scale-Revised.
