# Supplementary material for: Apathy as a Predictor for Conversion From Mild Cognitive Impairment to Dementia: A Systematic Review and Meta-Analysis of Longitudinal Studies
Source: J Geriatr Psychiatry Neurol. 2022 Apr 21;36(1):3–17. doi: 10.1177/08919887221093361 (PMC9755689; doi:10.1177/08919887221093361)
Supplement: Supplemental Material - Apathy as a Predictor for Conversion From Mild Cognitive Impairment to Dementia: A Systematic Review and Meta-Analysis of Longitudinal Studies [file sj-pdf-1-jgp-10.1177_08919887221093361.pdf]

Supplementary 1. Full search strategy.

Concept 1: Elderly people
aging/ or aged/ or "aged, 80 and over"/ or middle aged/ or (((aged* or old*) and (people or person or adult or man or men or woman or women or patient*)) or (elder* or ageing or aging or senior or older)).m_titl. or (((aged* or old*) and (people or person or adult or man or men or woman or women or patient*)) or (elder* or aging or ageing or senior or older)).ab.

Concept 2: MCI
"Mild cognitive impairment"/ or Memory disorders/ or ("memory disorder*" or "memory impairment*" or "memory loss*" or prodromal dementia or "mild cognitive impairment*" or preclinical dementia or predementia or "memory deficit*" or MCI or "cognitive deficit*" or "cognitive decline*" or "cognitive impairment*" or early stage dementia or "mild memory problem*" or "mild memory difficult*" or questionable dementia or cognitive deterioration).m_titl. or ("memory disorder*" or "memory impairment*" or "memory loss*" or prodromal dementia or "mild cognitive impairment*" or preclinical dementia or predementia or "memory deficit*" or MCI or "cognitive deficit*" or "cognitive decline*" or "cognitive impairment*" or early stage dementia or "mild memory problem*" or "mild memory difficult*" or questionable dementia or cognitive deterioration).ab.

Concept 3: BPSD
“behavioral and psychological symptoms in dementia,” “behavioral and psychological*,” “BPSD,” “neuropsychiatric symptoms,” hyperactive,” hyperactivity symptoms,” “psychosis,” psychotic,” “psychotic symptoms,” “affective symtoms,” “agitation,” ”hallucination,” “irritability,” “disinhibition,” “elation,” “motor disturbance,” “depression,” “anxiety,” “apathy,” “sleep,” “appetite,” “delusion,” “delirium,” “hallucination,” “behavioural symptoms[MESH]” OR “Delusions [MESH],” OR “Hallucinations [MESH]” OR “Psychomotor Agitation [MESH]” “OR Aggression [MESH]” OR “Dysphoria OR Anxiety[MESH]” OR “euphoria [MESH]” OR “Apathy[MESH]” OR “Irritable mood [MESH]” OR “Sleep disorders [MESH]” OR “Feeding and eating disorders [MESH]” OR “Aberrant vocalization” OR “Elation/ Disinhibition Irritability/lability” OR “Motor disturbances”

Concept 4: Prediction
"disease progression"/ or "early diagnosis"/ or "predictive value of tests"/ or prognosis/ or "sensitivity and specificity"/ or "severity of illness index"/ or (predict* or prognos* or early detect* or conversion or convert* or progression or early diagnos* or progressor or predictive accuracy or progres* or (sensitivity and specificity)).m_titl. or (predict* or prognos* or early detect* or conversion or convert* or progression or early diagnos* or progressor or predictive accuracy or progres* or (sensitivity and specificity)).ab.

Concept 5: Dementia
“Dementia” “dementia tests,” “Alzheimer` disease,” “Alzheimer’s dementia,” “Alzheimer*,” “vascular dementia,” “Lewy body dementia,” “Lewy Body disease,” “frontotemporal dementia,” “normal pressure hydrocephalus,” “mixed dementia,” “neurodegenerative disease,” “Huntington’s disease,” “Parkinson’s disease.”

Concept 6: Longitudinal studies
Cohort studies/ or follow up studies/ or prospective studies/ or longitudinal studies/ or retrospective studies/ or (cohort stud* or prospective stud* or follow up stud* or longitudinal stud* or retrospective stud* or prospective cohort stud* or community stud* or population-based stud*).m_titl. or (cohort

stud* or prospective stud* or follow up stud* or longitudinal stud* or retrospective stud* or prospective cohort stud* or community stud* or population-based stud*).ab.
