# Supplementary material for: Apathy as a Predictor for Conversion From Mild Cognitive Impairment to Dementia: A Systematic Review and Meta-Analysis of Longitudinal Studies
Source: J Geriatr Psychiatry Neurol. 2022 Apr 21;36(1):3–17. doi: 10.1177/08919887221093361 (PMC9755689; doi:10.1177/08919887221093361)
Supplement: Supplemental Material - Apathy as a Predictor for Conversion From Mild Cognitive Impairment to Dementia: A Systematic Review and Meta-Analysis of Longitudinal Studies [file sj-pdf-2-jgp-10.1177_08919887221093361.pdf]

Supplementary 2. Reasons for exclusion of full-text articles

First author and year of publication	Reason for exclusion
Banning 2020 (1)	Cross-sectional study design
Beaudreau 2013 (2)	Association between apathy and dementia only reported at baseline
Boccardi 2017 (3)	Cross-sectional study design
Brodaty 2012 (4)	Association between apathy and dementia not tested
Chan 2010 (5)	Association between apathy and dementia not tested
Creese 2019 (6)	Association between apathy and dementia not tested
David 2016 (7)	Association between apathy and dementia not tested
Delrieu 2015 (8)	Association between apathy and dementia not tested
Donovan 2014 (9)	Association between apathy and dementia not tested
Ferman 2013 (10)	Association between apathy and dementia not tested
Fernández-Martínez 2010 (11)	Association between apathy and dementia not tested
Forrester 2016 (12)	Association between apathy and dementia not tested
Franchini 2019 (13)	Association between apathy and dementia not tested
Grande 2020 (14)	Association between apathy and dementia not tested
Haroon Burhanullah 2020 (15)	Association between apathy and dementia not tested
Hsiung 2006 (16)	Association between apathy and dementia not tested
Johansson 2020 (17)	Association between apathy and dementia not tested
Kuhn 2019 (18)	Association between apathy and dementia not tested
Leoutsakos 2015 (19)	Cognitively normal sample
Liew 2018 (20)	Association between apathy and dementia not tested
Liew 2019 (21)	Association between apathy and dementia not tested
Mallo 2017 (poster)	Association between apathy and dementia not tested
Matsuoka 2019 (22)	Association between apathy and dementia not tested
Mauri 2012 (23)	Association between apathy and dementia not tested
Mortby 2017 (24)	Cross-sectional study design
Moylett 2019 (25)	Association between apathy and dementia not tested
Orso 2019 (26)	Imaging study
Palmer 2007 (27)	Association between apathy and dementia not tested

Peters 2008 (28)	Association between apathy and dementia not tested
Pocnet 2015 (29)	Association between apathy and dementia not tested
Rosenberg 2011 (30)	Association between apathy and dementia not tested
Royall 2020 (31)	Association between apathy and dementia not tested
Scaricamazza 2019 (32)	Cross-sectional study design
Seifan 2019 (33)	Association between apathy and dementia not tested
Skov Neergaard 2017 (34)	Association between apathy and dementia not tested
Soares 2017 (35)	Association between apathy and dementia not tested
Sugarman 2018 (36)	Same study population as other included study
Taragano 2018 (37)	Association between apathy and dementia not tested
Teng 2007 (38)	Association between apathy and dementia reported without standard deviation
Van der Mussele 2013 (39)	Cross-sectional study design
Van der Mussele 2014 (40)	Cross-sectional study design
Zhao 2021 (41)	Specific patient group; post-stroke

Reasons for exclusion of articles assessed in full-text.

References

1. Banning LCP, Ramakers IHGB, Köhler S, Bron EE, Verhey FRJ, de Deyn PP, et al. The Association Between Biomarkers and Neuropsychiatric Symptoms Across the Alzheimer's Disease Spectrum. The American Journal of Geriatric Psychiatry. 2020 Jul;28(7):735–44.

2. Beaudreau SA, Kaci Fairchild J, Spira AP, Lazzeroni LC, O'Hara R. Neuropsychiatric symptoms, apolipoprotein E gene, and risk of progression to cognitive impairment, no dementia and dementia: the Aging, Demographics, and Memory Study (ADAMS): Neuropsychiatric symptoms. Int J Geriatr Psychiatry. 2013 Jul;28(7):672–80.

3. Boccardi V, Conestabile Della Staffa M, Baroni M, Ercolani S, Croce MF, Ruggiero C, et al. Prevalence and Correlates of Behavioral Disorders in Old Age Subjects with Cognitive Impairment: Results from the ReGAl Project. J Alzheimers Dis. 2017;60(4):1275–83.

4. Brodaty H, Heffernan M, Draper B, Reppermund S, Kochan NA, Slavin MJ, et al. Neuropsychiatric symptoms in older people with and without cognitive impairment. J Alzheimers Dis. 2012;31(2):411–20.

5. Chan W-C, Lam LC-W, Tam CW-C, Lui VW-C, Chan SS-M, Chan W-M, et al. Prevalence of neuropsychiatric symptoms in chinese older persons with mild cognitive impairment-a population-based study. Am J Geriatr Psychiatry. 2010 Oct;18(10):948–54.

6. Creese B, Brooker H, Ismail Z, Wesnes KA, Hampshire A, Khan Z, et al. Mild Behavioral Impairment as a Marker of Cognitive Decline in Cognitively Normal Older Adults. The American Journal of Geriatric Psychiatry. 2019 Aug;27(8):823–34.

7. David ND, Lin F, Porsteinsson AP. Trajectories of Neuropsychiatric Symptoms and Cognitive Decline in Mild Cognitive Impairment. The American Journal of Geriatric Psychiatry. 2016 Jan;24(1):70–80.

8. Delrieu J, Desmidt T, Camus V, Sourdet S, Boutoleau-Bretonnière C, Mullin E, et al. Apathy as a feature of prodromal Alzheimer’s disease: an FDG-PET ADNI study. Int J Geriatr Psychiatry. 2015 May;30(5):470–7.

9. Donovan NJ, Amariglio RE, Zoller AS, Rudel RK, Gomez-Isla T, Blacker D, et al. Subjective Cognitive Concerns and Neuropsychiatric Predictors of Progression to the Early Clinical Stages of Alzheimer Disease. The American Journal of Geriatric Psychiatry. 2014 Dec;22(12):1642–51.

10. Ferman TJ, Smith GE, Kantarci K, Boeve BF, Pankratz VS, Dickson DW, et al. Nonamnestic mild cognitive impairment progresses to dementia with Lewy bodies. Neurology. 2013 Dec 3;81(23):2032–8.

11. M F-M, Molano A, Castro J, Zarranz JJ. Prevalence of neuropsychiatric symptoms in mild cognitive impairment and Alzheimer’s disease, and its relationship with cognitive impairment. Curr Alzheimer Res. 2010 Sep;7(6):517–26.

12. Forrester SN, Gallo JJ, Smith GS, Leoutsakos J-MS. Patterns of Neuropsychiatric Symptoms in Mild Cognitive Impairment and Risk of Dementia. The American Journal of Geriatric Psychiatry. 2016 Feb;24(2):117–25.

13. Franchini F, Musicco M, Ratto F, Storti G, Shofany J, Caltagirone C, et al. The LIBRA Index in Relation to Cognitive Function, Functional Independence, and Psycho-Behavioral Symptoms in a Sample of Non-Institutionalized Seniors at Risk of Dementia. J Alzheimers Dis. 2019;72(3):717–31.

14. Grande G, Vetrano DL, Mazzoleni F, Lovato V, Pata M, Cricelli C, et al. Detection and Prediction of Incident Alzheimer Dementia over a 10-Year or Longer Medical History: A Population-Based Study in Primary Care. Dement Geriatr Cogn Disord. 2020;49(4):384–9.

15. Burhanullah MH, Tschanz JT, Peters ME, Leoutsakos J-M, Matyi J, Lyketsos CG, et al. Neuropsychiatric Symptoms as Risk Factors for Cognitive Decline in Clinically Normal Older Adults: The Cache County Study. The American Journal of Geriatric Psychiatry. 2020 Jan;28(1):64–71.

16. Hsiung G-YR, Donald A, Grand J, Black SE, Bouchard RW, Gauthier SG, et al. Outcomes of Cognitively Impaired Not Demented at 2 Years in the Canadian Cohort Study of Cognitive Impairment and Related Dementias. Dement Geriatr Cogn Disord. 2006;22(5–6):413–20.

17. Johansson M, Stomrud E, Lindberg O, Westman E, Johansson PM, van Westen D, et al. Apathy and anxiety are early markers of Alzheimer’s disease. Neurobiology of Aging. 2020 Jan;85:74–82.

18. Kuhn E, Moulinet I, Perrotin A, La Joie R, Landeau B, Tomadesso C, et al. Cross-sectional and longitudinal characterization of SCD patients recruited from the community versus from a memory clinic: subjective cognitive decline, psychoaffective factors, cognitive performances, and atrophy progression over time. Alz Res Therapy. 2019 Dec;11(1):61.

19. Leoutsakos J-MS, Forrester SN, Lyketsos ConstantineG, Smith GS. Latent Classes of Neuropsychiatric Symptoms in NACC Controls and Conversion to Mild Cognitive Impairment or Dementia. JAD. 2015 Sep 9;48(2):483–93.

20. Liew TM, Yu J, Mahendran R, Ng T-P, Kua E-H, Feng L. Neuropsychiatric and Cognitive Subtypes among Community-Dwelling Older Persons and the Association with DSM-5 Mild Neurocognitive Disorder: Latent Class Analysis. J Alzheimers Dis. 2018;62(2):675–86.

21. Liew TM. Symptom Clusters of Neuropsychiatric Symptoms in Mild Cognitive Impairment and Their Comparative Risks of Dementia: A Cohort Study of 8530 Older Persons. Journal of the American Medical Directors Association. 2019 Aug;20(8):1054.e1-1054.e9.

22. Matsuoka T, Ismail Z, Narumoto J. Prevalence of Mild Behavioral Impairment and Risk of Dementia in a Psychiatric Outpatient Clinic. Abbate C, editor. JAD. 2019 Jul 23;70(2):505–13.

23. Mauri M, Sinforiani E, Zucchella C, Cuzzoni MG, Bono G. Progression to dementia in a population with amnestic mild cognitive impairment: clinical variables associated with conversion. Funct Neurol. 2012 Mar;27(1):49–54.

24. Mortby ME, Burns R, Eramudugolla R, Ismail Z, Anstey KJ. Neuropsychiatric Symptoms and Cognitive Impairment: Understanding the Importance of Co-Morbid Symptoms. J Alzheimers Dis. 2017;59(1):141–53.

25. Moylett S, Price A, Cardinal RN, Aarsland D, Mueller C, Stewart R, et al. Clinical Presentation, Diagnostic Features, and Mortality in Dementia with Lewy Bodies. J Alzheimers Dis. 2019;67(3):995–1005.

26. Orso B, Mattei C, Arnaldi D, Massa F, Serafini G, Plantone D, et al. Clinical and MRI Predictors of Conversion From Mild Behavioural Impairment to Dementia. The American Journal of Geriatric Psychiatry. 2020 Jul;28(7):755–63.

27. Palmer K, Berger AK, Monastero R, Winblad B, Backman L, Fratiglioni L. Predictors of progression from mild cognitive impairment to Alzheimer disease. Neurology. 2007 May 8;68(19):1596–602.

28. Peters KR, Rockwood K, Black SE, Hogan DB, Gauthier SG, Loy-English I, et al. Neuropsychiatric Symptom Clusters and Functional Disability in Cognitively-Impaired-Not-Demented Individuals. The American Journal of Geriatric Psychiatry. 2008 Feb;16(2):136–44.

29. Pocnet C, Antonietti J-P, Donati A, Popp J, Rossier J, von Gunten A. Behavioral and psychological symptoms and cognitive decline in patients with amnestic MCI and mild AD: a two-year follow-up study. Int Psychogeriatr. 2015 Aug;27(8):1379–89.

30. Rosenberg PB, Mielke MM, Appleby B, Oh E, Leoutsakos J-M, Lyketsos CG. Neuropsychiatric symptoms in MCI subtypes: the importance of executive dysfunction. Int J Geriat Psychiatry. 2011 Apr;26(4):364–72.

31. Royall DR, Palmer RF. δ scores predict multiple neuropsychiatric symptoms. Int J Geriatr Psychiatry. 2020 Nov;35(11):1341–8.

32. Scaricamazza E, Colonna I, Sancesario GM, Assogna F, Orfei MD, Franchini F, et al. Neuropsychiatric symptoms differently affect mild cognitive impairment and Alzheimer's disease patients: a retrospective observational study. Neurol Sci. 2019 Jul;40(7):1377–82.

33. Seifan A, Ganzer CA, Ryon K, Lin M, Mahmudur R, Adolfo H, et al. Detecting Non-cognitive Features of Prodromal Neurodegenerative Diseases. CAS. 2019 Apr 22;11(4):242–9.

34. Skov Neergaard J, Dragsbæk K, Christiansen C, Asser Karsdal M, Brix S, Henriksen K. Objective Cognitive Impairment and Progression to Dementia in Women: The Prospective Epidemiological Risk Factor Study. J Prev Alzheimers Dis. 2017;4(3):194–200.

35. Soares WB, dos Santos EB, Bottino CM de C, Elkis H. Psychotic symptoms in older people without dementia from a Brazilian community-based sample: A seven years' follow-up. Ginsberg SD, editor. PLoS ONE. 2017 Jun 16;12(6):e0178471.

36. Sugarman MA, Alosco ML, Tripodis Y, Steinberg EG, Stern RA. Neuropsychiatric Symptoms and the Diagnostic Stability of Mild Cognitive Impairment. JAD. 2018 Mar 27;62(4):1841–55.

37. Taragano FE, Allegri RF, Heisecke SL, Martelli MI, Feldman ML, Sánchez V, et al. Risk of Conversion to Dementia in a Mild Behavioral Impairment Group Compared to a Psychiatric Group and to a Mild Cognitive Impairment Group. J Alzheimers Dis. 2018;62(1):227–38.

38. Teng E, Lu PH, Cummings JL. Neuropsychiatric symptoms are associated with progression from mild cognitive impairment to Alzheimer's disease. Dement Geriatr Cogn Disord. 2007;24(4):253–9.

39. Van der Mussele S, Le Bastard N, Vermeiren Y, Saerens J, Somers N, Mariën P, et al. Behavioral symptoms in mild cognitive impairment as compared with Alzheimer's disease and healthy older adults: Behavioral symptoms in mild cognitive impairment. Int J Geriatr Psychiatry. 2013 Mar;28(3):265–75.

40. Van der Mussele S, Mariën P, Saerens J, Somers N, Goeman J, De Deyn PP, et al. Behavioral syndromes in mild cognitive impairment and Alzheimer's disease. J Alzheimers Dis. 2014;38(2):319–29.

41. Zhao J, Jin X, Chen B, Fu C, Ji S, Shen W, et al. Apathy symptoms increase the risk of dementia conversion: a case- matching cohort study on patients with post- stroke mild cognitive impairment in CHINA. Psychogeriatrics. 2021 Mar;21(2):149–57.
